# The hidden risk of round numbers and sharp thresholds in clinical practice

**DOI:** 10.1038/s41746-025-02079-y

**Published:** 2025-11-21

**Authors:** Benjamin J. Lengerich, Rich Caruana, Mark E. Nunnally, Manolis Kellis

**Affiliations:** 1 Massachusetts Institute of Technology, Cambridge, MA USA; 2 Broad Institute of MIT and Harvard, Cambridge, MA USA; 3 University of Wisconsin, Madison, WI USA; 4 Microsoft Research, Redmond, WA USA; 5 NYU Langone Health, New York City, NY USA

**Keywords:** Computational biology and bioinformatics, Diseases, Health care, Mathematics and computing, Medical research

## Abstract

Clinical decision-making often simplifies continuous risk data into discrete levels using round-number thresholds. These simplifications can distort risk assessments. To systematically uncover these distortions, we develop an interpretable machine learning model that identifies anomalies caused by threshold-based practices. Through simulations, real-world data, and longitudinal studies, we demonstrate how round-number thresholds can lead to discontinuities and counter-causal paradoxes in mortality risk. Despite advances in medicine, these anomalies persist, underscoring the need for dynamic and nuanced risk assessment methods in healthcare. Our findings suggest continuous reassessment of clinical protocols, especially in critical settings like intensive care, to improve patient outcomes by aligning thresholds with the continuous nature of risk.

## Introduction

**Fig. 1:**
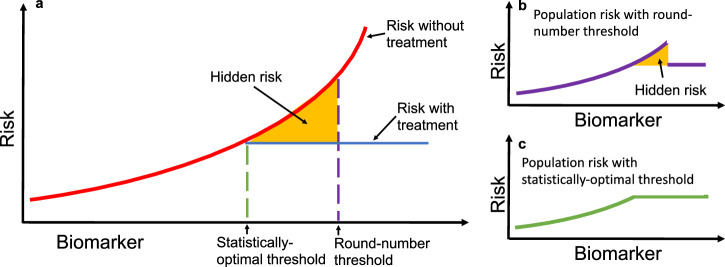
Hidden risk from round-number thresholds in biomarker-based decisions. **a** A hypothetical biomarker is used to guide treatment, with patient risk on the vertical axis. The solid red line shows untreated risk, and the solid blue line shows treated risk. The dashed purple line marks a round-number threshold, while the dashed green line represents the statistically optimal threshold that minimizes risk. The yellow shaded area highlights excess risk incurred when decisions are based on the round-number threshold instead of the optimal threshold. **b** Applying a round-number threshold produces excess risk due to undertreatment or overtreatment. **c** Using the statistically optimal threshold ensures continuous and monotonic risk reduction, eliminating this excess risk. In practice, the optimal threshold may lie above or below the round-number threshold, leading to systematic under- or overtreatment.

Clinical practice requires consistency of application and ease of implementation. Based on these requirements, guidelines for clinical practice are often presented as round-number thresholds to facilitate uniform decision-making. For example, both the Acute Physiology and Chronic Health Evaluation (APACHE II^1^) and Sepsis-related Organ Failure Assessment (SOFA^2^) calculators for assessing risk in ICUs set a threshold for serum creatinine at 3.5 mg/dL. While this practice of using memorable round-number thresholds simplifies clinical decision-making, it can also distort risk assessments and lead to suboptimal patient outcomes. For example, when a threshold for deciding treatment is misaligned from statistical optimality (e.g., due to a preference for a memorable round-number), there is excess risk conferred to the patients who could have been treated under a more statistically-optimal threshold (Fig. 1).

Identifying these suboptimal outcomes has historically been challenging due to ubiquitous confounding in observational medical data. Instead of attempting to perfectly de-confound observational data, our approach reframes this pervasive confounding as an analytical opportunity. By recognizing that threshold-induced treatment decisions create characteristic artifacts in observed population risks, we can identify areas of surprising risk and potentially suboptimal treatment decisions without requiring perfect deconfounding of treatment.

**Fig. 2:**
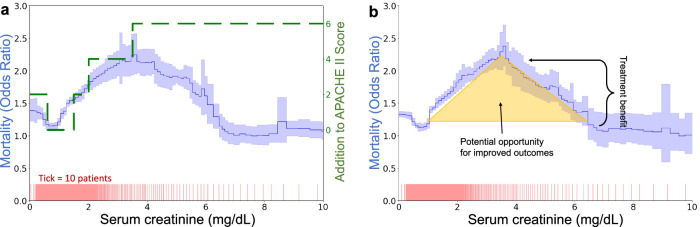
Inflection points in data-driven mortality risk curves reflect common round-number thresholds used for clinical decision-making. **a** Divergence between APACHE II risk categorization (green dashed line) and actual risk observed in MIMIC-IV (solid blue line with 95% confidence intervals shaded). While APACHE II shows monotonically increasing risk, the data-driven curve peaks at 3.5 mg/dL. **b** The shape of the creatinine curve suggests opportunities for enhanced care, as decreasing risk at very high levels implies that treatments effective for the sickest patients might also benefit those at medium risk.

Our approach is exemplified in Fig. 2, which demonstrates the discrepancies between theoretical predictions of risk based on established models like APACHE II and actual outcomes from real-world data such as the MIMIC-IV dataset. Contrary to model predictions that suggest a consistent increase in risk with higher serum creatinine levels, actual data exhibit a peak risk at a certain threshold followed by a decrease in risk as serum creatinine continues to climb. This unexpected pattern suggests a potential misalignment between current clinical interventions and the actual needs of patients, i.e., if the treatment that lowers mortality above 3.5 mg/dL were more consistently applied at lower creatinine levels, would it further smooth and flatten the risk curve and reduce mortality in the region around 2.0–4.5 mg/dL? To systematically uncover these areas of surprising risk, we develop glass-box machine learning (ML) techniques that uncover artifacts of threshold-based decision making. Based on the glass-box ML model, we develop statistical tests for two types of threshold-induced artifacts: discontinuous risk, commonly observed at round-number thresholds, and paradoxical risk, where successful treatments unexpectedly lower the risk of higher-risk patients to below that of untreated lower-risk patients. Our analysis is structured in four parts:

1. Real-world assessment: we assess real-world evidence by analyzing a dataset of mortality in hospitalized pneumonia patients, building a classification system for threshold effects in clinical settings.
2. Simulation analysis: motivated by these observations in real-world evidence, we develop a simplified model of treatment benefits, establishing a causal explanation for each type of statistical artifact.
3. Glass-box ML model: based on the classification system above, we develop a glass-box ML approach to systematically identify statistical artifacts from the observed population risk alone.
4. Longitudinal study: we apply the glass-box ML approach to the widely-used MIMIC dataset, collected over several decades, to understand how treatment effects have changed over time.

## Results

### Mortality risks of pneumonia patients exhibit surprising statistical patterns

To investigate and categorize the real-world impact of clinical decision-making thresholds, we analyzed mortality risks using real-world data to find surprising statistical patterns in patient outcomes. We conducted this exploratory data analysis by training generalized additive models (GAMs)^3^ to estimate mortality risk as the additive effects of individual risk factors. The GAMs demonstrated high predictive accuracy, comparable to fully-connected neural networks and XGBoost baselines (Supplementary Table 2), and provided high-resolution, nonlinear, component functions that can be sequentially analyzed. Our analysis used the 1989 MedisGroups Comparative Hospital Database (MCHD) pneumonia dataset^4^. We see two repeated statistical patterns that routinely indicate underlying treatment effects: (1) *discontinuities* in risk, and (2) counter-causal *non-monotonicities* in risk.

**Fig. 3:**
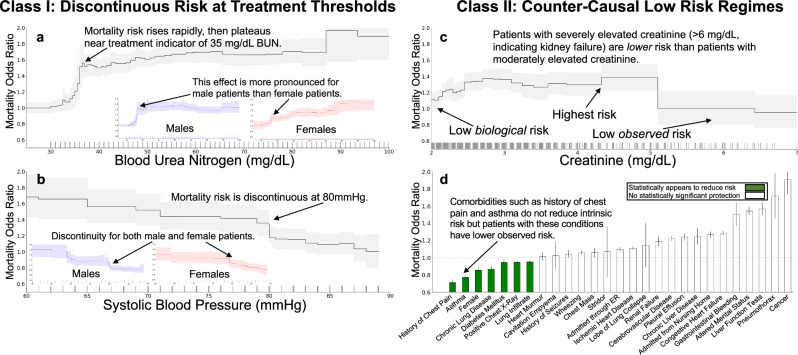
Statistical artifacts of confounding appear in the mortality risk of patients with pneumonia as two broad categories: discontinuities and counter-causal effects. In all plots, we show the effect of each variable on patient mortality risk (with 95% confidence intervals shaded) after correcting for all other observable risk factors using a generalized additive model. Each tick on the horizontal axis denotes ten observed patients. Discontinuities arise from behavior influenced by discrete round-number thresholds, including **a** a flattening in risk at a BUN of 35 mg/dL and **b** a discontinuous drop in risk at a systolic blood pressure of 80 mmHg. Counter-causal trends occur when intrinsically high-risk features are predicted as low-risk due to effective treatment, including **c** serum creatinine above 5 mg/dL and **d** chronic comorbidities, where patients often receive more aggressive care. Differences by sex are shown in (**c**), illustrating population stratification in risk profiles.

Discontinuities in risk often reveal threshold-based treatment effects in clinical data. Blood urea nitrogen (BUN) exhibits a discontinuous association with mortality risk (Fig. 3a), with a non-monotone structure. Intrinsic (i.e., untreated) risk is likely to increase smoothly with increasing BUN, so this discontinuous population risk suggests that different treatments are being applied at different BUN levels. Lower levels of BUN are associated with survival; however, there is a rapid rise in mortality risk from 30–40 mg/dL followed by a plateau in risk from 40–80 mg/dL. The pattern of risk at BUN of 40 mg/dL is stronger for male patients than female patients, with the risk for female patients rising continuously from 30–50 mg/dL, suggesting a stronger adherence to threshold-based decisions for male patients than for female patients. Similarly, systolic blood pressure shows a discontinuous association with mortality risk (Fig. 3b). There is a sharp discontinuous rise in risk as systolic blood pressure decreases below 80 mmHg, which suggests adherence to a threshold-based protocol.

Counter-causal non-monotonicities reveal that aggressive treatment can have a stronger positive effect than the deleterious effect of intrinsic biological risk. For example, elevated serum creatinine above 5 mg/dL (Fig. 3c) is associated with increased survival (lower mortality risk) of pneumonia patients. This effect is measured after correcting for all other patient risk factors recorded in the dataset and contradicts medical causality, as serum creatinine above 5 mg/dL indicates advanced renal dysfunction^5^. The correspondence between this counter-causal effect and a round-number threshold suggests that intervening factors, including aggressive treatment, are influenced by this threshold. Unfortunately, purely data-driven AI protocols could de-prioritize care for these patients who were effectively treated and might even use this extreme region as a therapeutic target (i.e., withhold treatment to keep serum creatinine high, or even worse, suggest treatments to increase serum creatinine).

Chronic comorbidities also produce paradoxical statistical effects. For example, the odds ratio of mortality in pneumonia patients is *reduced* by more than 30% with a history of chest pain, 20% with a history of asthma, and 18% by a chronic lung disease (Fig. 3d). These counter-causal effects are robustly estimated and not sensitive to the model class or algorithm used for estimation, suggesting that risk models trained on these real-world datasets could systematically underestimate mortality risk (effective risk < underlying risk) for patients with prior comorbidities. In real-world data, observed effective risk implicitly combines underlying risk with the impact of interventions (including unobserved quantities such as perceived urgency by both the patient and the clinician). As a result, the effective risk for patients with chronic comorbidities is often lower than the underlying risk for the same patients; hence, data-driven AI models can systematically underestimate the underlying risk associated with chronic comorbidities.

### A model of how threshold-guided interventions generate statistical artifacts in risks

**Fig. 4:**
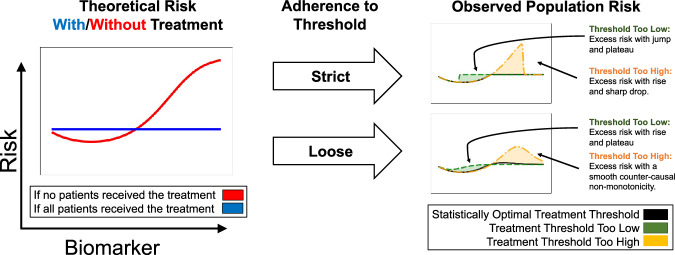
Confounding from treatment effects produces characteristic shapes in population risk. Based on simulations, we depict intrinsic risk for treated and untreated patients (left), adherence of treatment decisions to a threshold (middle), and observed risk (right). The optimal treatment threshold is where untreated risk crosses above treated risk; when protocols are misaligned, observed risk includes excess risk. The flat “with treatment” curve assumes a simplified, short-term view of treatment effects, where treatment applied too early may increase risk, and treatment applied at or after the optimal threshold can flatten high-risk regions if highly effective. For alternative assumptions of treatment benefits and resulting artifacts, see Supplementary Fig. 2.

To build a model for systematically identifying and understanding the potential impacts of confounding from threshold-guided clinical practice, we first build a simplified model of how risk curves are generated from simulated treatment benefits and protocols in a controlled setting (Fig. 4 and Supplementary Fig. 2). We study eight simulation cases; for each simulation case, we select one of four treatment benefit curves: flattens risk, limits risk, reduces biomarker, or has constant benefit (the left column in Fig. 4 and Supplementary Fig. 2) and one of two treatment probability distributions: strict adherence to a biomarker treatment threshold, or loose guidance based on a biomarker threshold (the middle column in Fig. 4 and Supplementary Fig. 2). These underlying risk and treatment curves combine to produce a population risk—the right column in Fig. 4 and Supplementary Fig. 2. The shape of the observed population risk (right column) is influenced by the shape of the intrinsic risk prior to treatment (left column) and the treatment decisions. From this simple causal explanation, we are able to generate many statistical artifacts in observed population risk curves, all of which can be categorized into: (1) *discontinuities* and (2) *non-monotonicities* in risk.

Threshold-based decisions cause sharp cutoffs in real-world risk. In some cases, these cutoffs produce discontinuous risk profiles, suggesting that the intervention could be more precisely distributed or dosed to achieve statistical optimality. For example, strict adherence to a threshold that is too high (Fig. 4, yellow) results in observed population risk that includes excess risk below the treatment threshold where treatment should, but is not, being given, with a sharp, discontinuous drop in risk at the honored treatment threshold.

When the threshold is only loosely adhered to (possibly in a multi-factor decision list), the discontinuity turns into a non-monotonicity. As medicine becomes increasingly precise, multi-faceted, AI-supported, and personalized, we expect to see risk curves smooth out round-number biases and flatten observed risks—we later discuss an example (Fig. 6d) where recent improvements in clinical decision-making have already smoothed and flattened risk curves.

### Using glass-box ML to systematically find statistical artifacts in risks

Based on the morphology of these two consistent types of artifacts in population risk (discontinuities and non-monotonicities), we develop a glass-box ML approach to systematically search for these two characteristic surprises. Our approach first uses GAMs to decompose risk into additive component functions of individual risk factors and then finds regions of discontinuities and/or non-monotonicities in each component.

**Fig. 5:**
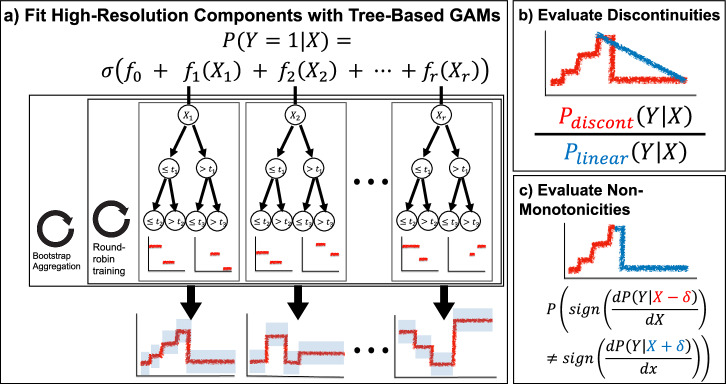
Summary of our approach to estimate outcome probabilities and automatically find statistical artifacts of clinical practice. **a** Sketch of the tree-based GAM trained by InterpretML, which models the logit probability of outcome *Y* as an additive function of individual features of *X*. This model uses round-robin training to split information between individual component functions and bootstrap aggregation for confidence intervals. **b** For each component function and each threshold therein, we evaluate the likelihood of a discontinuity by comparing the probability of observed data under the discontinuous component function against a locally-linear version of the component function. **c** For each component function and each threshold therein, we evaluate the likelihood of a non-monotonicity by changepoint detection to the nonzero slopes of the signs of the component function.

To automate the search for discontinuities and non-monotonicities within each component, we define two statistical tests on the likelihood of the shape of a component (Fig. 5). We use boosted decision trees to train the GAM models because these have been shown to have excellent accuracy and high resolution in the learned feature functions. Moreover, the ability of trees to learn “jumps” in feature functions aids detection of discontinuities and non-monotonicities. For each component function *f*_*r*_ estimated by a tree-based GAM, we have a set of thresholds *t*_*r*_ and associated probability densities *y*_*r*_. To test for a discontinuity at threshold *t*_*r*_, we compare the probability of observed outcomes under the discontinuous threshold-based component function *f*_*r*_ against the probability of observed outcomes under a linearized version of *f*_*r*_ (Fig. 5b). To test for a non-monotonicity at threshold *t*_*r*_, we apply changepoint detection^6^ to the sign of non-zero slopes of the component function (Fig. 5c). For details of these tests, see “Methods”.

### Threshold-based artifacts reveal how clinical practice has improved over years of refinement

To study how clinical practice and threshold-based biases effects have changed over years of protocol refinement, we compare the statistical artifacts of clinical practice in three versions of the Multiparameter Intelligent Monitoring in Intensive Care (MIMIC) dataset^7–9^. These datasets were collected over three decades of management of intensive care units (ICU) at a single hospital, providing high-quality snapshots of medical practice and outcomes over many years. We find several likely examples in which the harmful impact of round-number threshold effects has been ameliorated over the years of refinement in practice. However, counter-causal and threshold-driven effects are still strong and robustly estimated by data-driven risk models. Finally, we validate the hypothesis of treatment effects driving these statistical artifacts by identifying treatments recorded in MIMIC-IV^9^ that correspond to counterfactual changes in risk and de-confound the effects of treatment from the underlying biological risk. Overall, we find that the statistical artifacts of clinical practice are associated with observable treatment patterns, typically based on clinically-meaningful and round-number thresholds, and that while these effects have diminished over time, they are still routinely and robustly identified to impact patient mortality risk. Here, we study four examples of these effects.

**Fig. 6:**
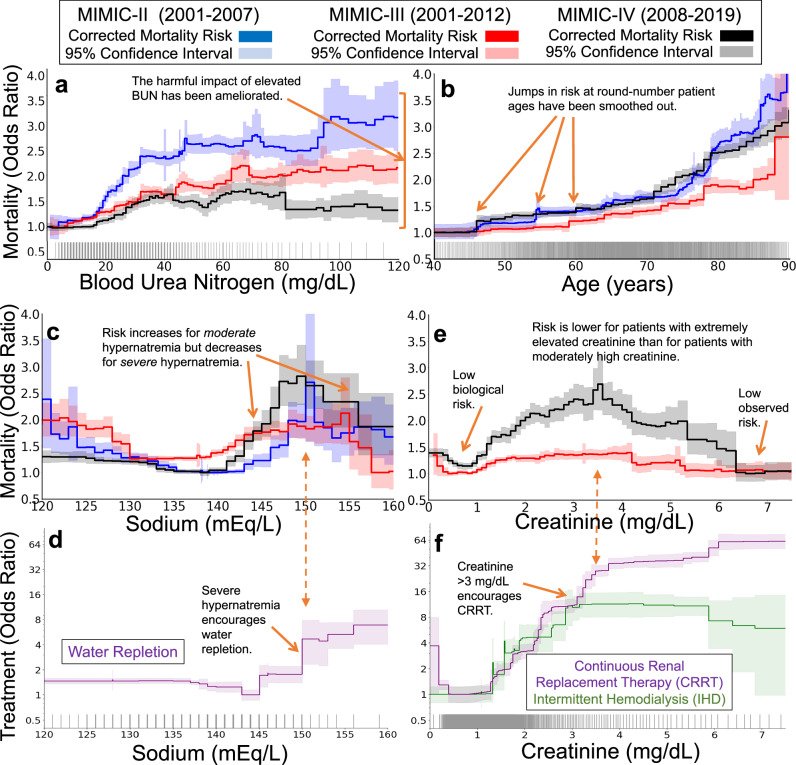
Clinical practice has changed and produced visible changes in mortality risk curves over time. The three datasets (MIMIC-II, MIMIC-III, and MIMIC-IV) span three decades of intensive care at a single hospital system, allowing comparisons of the effects of clinical practice over time. The top two rows show mortality risk associated with each risk factor (after correcting for all other observable factors of patient risk, 95% confidence intervals shaded). The bottom row shows treatment likelihood associated with each risk factor in MIMIC-IV (with 95% confidence intervals shaded). Each tick along the horizontal axes denotes ten patients. **a** An example of successful improvement in clinical practice: the harmful mortality risk attributable to elevated blood urea nitrogen has been ameliorated over time, reducing from large impact in the early 2000s (blue) to a small impact in the 2010s (black). **b** A second example of successful improvement in clinical practice: the mortality risk attributable to patient age has been smoothed. **c**, **d** An example of a persistent counter-causal effect. In all three datasets, the mortality risk attributable to serum sodium (**c**) decreases at 150 mEq/L. This counter-causal change in mortality risk is associated with an increase in water and electrolyte replacement (Dextrose 5%, Free Water, Potassium Phosphate, Sterile Water, Sodium Chloride 0.45%) (**d**). **e**, **f** As treatment effectiveness has improved over time, some risks have become even more counter-causal. For example, in the most recent version of MIMIC, the mortality risk associated with serum creatinine is maximized for moderately elevated creatinine and decreases sharply for severely elevated creatinine (**e**). This decrease in risk is associated with increased use of continuous renal replacement therapy (**f**), suggesting potential for improvements in clinical practice to spread the benefits of this care to a broader set of patients with lower creatinine levels.

As a first example, we see that harmful effects associated with blood urea nitrogen (BUN) have been ameliorated. In the oldest version of MIMIC, BUN strongly influenced mortality risk (Fig. 6a), with a sharp rise in risk from 20–35 mg/dL followed by a plateau from 35–100 mg/dL. In more recent datasets, this sharp rise has diminished, likely due to advancements in treatment protocols and the incorporation of BUN into multi-factor risk measures^10^.

As a second example, we see that discontinuous effects of age have been smoothed. Patient age exhibits a discontinuous impact on mortality rates (Fig. 6b), but these effects have smoothed over time. In particular, discontinuous rises in risk at 50, 55, and 60 years of age have been smoothed. While the impact of these round-number ages has reduced, age 80 continues to confer increased risk in all 3 three datasets, suggesting that changes to the treatment of elderly patients could reduce this risk and flatten the discontinuity at 80.

As a third example, we see that counter-causal effects of serum sodium have endured. As expected from medical intuition regarding hydration, the healthy range of 135–145 mEq/L serum sodium is associated with low mortality risk in all three datasets (Fig. 6c). On either side of this healthy range, the probability of mortality increases, rising rapidly for hyponatremia below 135 mEq/L and hypernatremia above 145 mEq/L. However, in all three datasets, this medical intuition is broken at 150 mEq/L: the probability of mortality declines for extremely elevated serum sodium. This threshold of 150 mEq/L is an indicator of moderate hypernatremia^11^ and is associated with increased water and electrolyte repletion (Fig. 6d). As a result of aggressive treatment of severe hypernatremia and under-treatment for mild hypernatremia^12^, the probability of mortality is *lower* for patients with serum sodium above 155 mEq/L than for patients with serum sodium of 145 mEq/L. This effect is robustly supported by statistical evidence, persistent over decades of care, and clinically meaningful—any accurate model trained on these datasets has learned that severe hypernatremia is lower risk than moderate hypernatremia, so applying these data-driven models in clinical practice could divert treatments away from the group of patients who would benefit *most* from treatment.

Finally, as a fourth example, we see that deleterious counter-causal effects of serum creatinine have strengthened. Elevated levels of serum creatinine indicate kidney dysfunction^13^; however, this biomarker is not strongly associated with mortality at extremely high values (Fig. 6e). Instead, in MIMIC-IV, the highest risk is observed for patients with moderate serum creatinine in the range 3–5 mg/dL. This counter-causal effect has strengthened between MIMIC-III and MIMIC-IV. This surprisingly non-monotone risk is linked to strong changes in treatment: the use of continuous renal replacement therapy (CRRT) sharply increases at both 3 mg/dL and 6 mg/dL (Fig. 6f). These patterns suggest that such extra monitoring for kidney dysfunction and corresponding interventions like CRRT have successfully reduced the mortality risk of patients with severely-elevated creatinine below the risk of patients with healthy levels of creatinine, leaving an opportunity to improve the outcomes of more patients by more widespread use of these interventions.

## Discussion

This study reveals significant biases introduced by threshold-based decision-making in clinical settings. Our analyses-spanning simulations, real-world data, and longitudinal studies-demonstrate how round-number thresholds can lead to discontinuities and counter-causal paradoxes in risk assessments. Using an interpretable machine learning model, we systematically identified these statistical artifacts and linked them to treatments.

These findings highlight the need to periodically reassess clinical guidelines to ensure alignment between underlying biological risk and real-world risk. A challenge is to balance the continuous nature of risk against the required simplicity and practicality of clinical scoring systems (e.g., APACHE II and SOFA). Clinicians rely on these simplified systems precisely because they facilitate quick and consistent evaluations in high-pressure clinical environments. Our objective is not to advocate for replacing these scores outright, but rather to quantify the hidden risks introduced by rigid, round-number thresholds. By identifying these statistical artifacts, our approach offers complementary insights to support clinicians and guideline developers in refining existing practice. Importantly, our model incorporates multiple biomarkers and demographic variables simultaneously, capturing part of the multifactorial nature of clinical practice, while acknowledging that many patient-specific considerations remain beyond the scope of available data.

Moreover, while applying AI to refine treatment guidelines may appear to be a straightforward prediction problem, our study uncovers a hidden danger: AI tools trained on observational data routinely misjudge patient risk by confusing inherent low risk with the reduced risk achieved through effective, routine treatments. Consequently, data-driven models can underestimate the true risk for patients who regularly receive effective treatment, potentially leading to insufficient care recommendations. To counteract this, AI models must be designed to recognize and adjust for such biases, favoring transparency and interpretability over black-box predictive models. Our approach applies this perspective by using glass-box models that directly visualize and quantify opportunities for improved medical practice.

This study has limitations, including reliance on ICU datasets that may not fully represent other medical settings, and use of a simplified model of treatment decisions to visualize characteristic artifacts. Establishing definitive causal links between threshold practices and patient outcomes will require prospective studies in various medical environments. Moreover, the interpretability and robustness of findings depend upon the quality and representativeness of available datasets. Some apparent discontinuities may also reflect sparse data in certain regions of the predictor space. Our bootstrap resampling procedure highlights these areas of high uncertainty, reducing the risk of misinterpreting such artifacts as genuine effects. In addition, effective clinical risk post-treatment can be lower than the underlying biological risk, which may lead models to underestimate risk for patients with chronic comorbidities, such as those with chronic kidney disease. Future research should explore broader contexts beyond ICU datasets and diverse patient populations, as well as further developing models that integrate ongoing, dose-dependent treatments into prognostic risk assessments, as recently explored in pediatric critical care^14^, and explicitly quantify cost–benefit considerations.

In conclusion, although threshold-based decision-making simplifies the management of complex clinical data, it introduces biases that can compromise patient care. Traditional analyses have focused on correcting for all confounding before estimating the effects of such simplification. In contrast, we have shown that the characteristic patterns of confounding can be a valuable tool for exploratory data analysis. By using advanced glass-box ML models to reveal discontinuities and non-monotonicities in risk, we can identify and correct these biases, facilitating more precise and personalized clinical decision-making. As AI becomes increasingly integrated in healthcare, it is paramount that these tools are designed to expose and correct biases, rather than silently propagate such biases.

## Methods

### Datasets

We use four datasets in this study: the MedisGroups Comparative Hospital Database (MCHD) and three versions of the Multiparameter Intelligent Monitoring in Intensive Care (MIMIC).

The MCHD pneumonia dataset^4^ contains information on inpatients from 78 hospitals in 23 states in the US between July 1987 and December 1988. The MCHD contains over 250 pieces of clinical information that include patient demographic characteristics, history and physical examination findings, and laboratory and radiological results, from which 46 variables were selected^4^ with known or postulated association with mortality in patients with community-acquired pneumonia. We used patient data that were collected during the first 48 h of hospitalization.

The MIMIC dataset is a high-quality collection of thousands of hospitalizations at the Beth Israel Deaconess Medical Center. This dataset has been provided in several iterations and used by thousands of researchers to estimate mortality risk models. We use three versions of this dataset: MIMIC-II^8^ (24,509 hospitalizations), MIMIC-III^7^ (27,349 hospitalizations), and MIMIC-IV^9^ (76,540 hospitalizations). To standardize the datasets between the versions, we use only patients admitted to an ICU and select only the intersection of patient risk factors available in at least 2 datasets.

### Model estimation

We use generalized additive models (GAMs)^3^ trained with boosted decision trees^15,16^ using the open-source package InterpretML^17^. GAMs are ideal to build glass-box models of patient risk because: (1) GAMs can be precisely decomposed into risk curves of single variables for interpretation, (2) the flexible component functions allow risk curves of any shape without any implicit preferences, (3) many treatment protocols and clinical decisions (which sum multiple sources of evidence) are inherently additive models (e.g., SAPS II^18^, APACHE II^19^), (4) GAMs provide the ability to edit the model^20^ and reason about changes to univariable treatment protocol thresholds. We use boosted trees to train GAMs because tree-based models are scale invariant, allowing features to be represented in their original natural units (including Boolean, nominal, ordinal, or continuous-valued attributes) without biases of pre-processing. Tree-based models can estimate discontinuities that smoother models, such as spline-based GAMs and neural networks, miss. The Explainable Boosting Machine (EBM) GAMs in InterpretML are particularly useful for healthcare because they use a round-robin fitting procedure that helps ensure hidden effects are observable in the estimated model. Finally, InterpretML uses bootstrap resampling^21^ to reduce variance and make the learned models easier to interpret, and also to estimate confidence intervals that help distinguish true from spurious detail in the learned response curves. We use default hyperparameters with one exception: we increase the number of rounds of bootstrap sampling from 10 to 100 (following the convention suggested by the algorithm designers^22^), in order to have better uncertainty estimates. We benchmark model accuracy against baselines (Supplementary Table 2), including several versions of XGBoost and neural networks to measure accuracy.

### Automated detection of discontinuities

Let *r* be the component of interest, with component function *f*_*r*_ estimated by the tree-based GAM defining a set of thresholds *t*_*r*_ and associated outcome odds *y*_*r*_. We construct a locally-linear version of *f*_*r*_:

1 $$\begin{array}{l}\tilde{{f}_{r}}(x)=\left\{\begin{array}{ll}{f}_{r}(x)\quad &x < {t}_{r,j-1}\\ {y}_{r,j-1}+\frac{x-{t}_{r,j-1}}{{t}_{r,j+1}-{t}_{r,j-1}}({y}_{r,j+1}-{y}_{r,j-1})\quad &{t}_{r,j-1}\le x < {t}_{r,j+1}\\ {f}_{r}(x)\quad &x\ge {t}_{r,j+1}\end{array}\right.\end{array}$$

To evaluate a discontinuity at threshold *t*_*r*,*j*_, we define a test statistic *T*_*d*_ which compares the log-likelihood of the discontinuous component against the log-likelihood of the linearized component:

2 $${T}_{d}(r,t| X,Y,{f}_{r})=\ell ({f}_{r}| X,Y)-\ell (\tilde{{f}_{r}}| X,Y)$$

3 $$=\log P(Y| X,{f}_{r})-\log P(Y| X,\tilde{{f}_{r}})$$

4 $$=\sum _{i}\,\text{sign}\,({Y}_{i})\left(\log {f}_{r}({x}_{r})-\log{\tilde{{f}}_{r}}({x}_{r})\right)$$

where *i* indexes observed samples, and the final equality holds by additivity of component functions in the GAM. This test statistic estimates the likelihood of a discontinuity at each threshold; the bootstrap provides confidence intervals.

### Automated detection of non-monotonicities

**Fig. 7:**
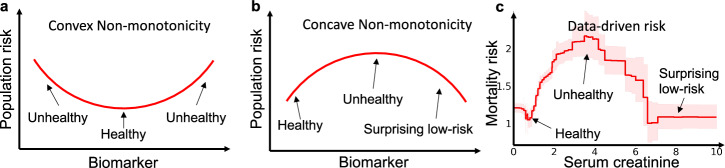
In risk graphs, convex non-monotonicities often indicate normal healthy regions of risk, while concave non-monotonic often indicate hidden confounding. **a** Many biomarkers exhibit intrinsic risk profiles (prior to treatment) that are convex because there is a normal, healthy region in the middle or left of the graph, with higher intrinsic risk for patients that are too low or too high. **b** Counter-causal non-monotonicities caused by treatment effects that are not well aligned with the optimal treatment thresholds, however, often exhibit a surprising concave data-driven risk profile where risk peaks at an intermediate value (or set of values) of the biomarker and then drops as the biomarker increases beyond the treatment threshold(s). **c** Mortality risk in the pneumonia dataset demonstrates both of these behaviors, with a healthy convex non-monotonicity centered at 1 mg/dL and a surprising concave non-monotonicity centered at 3.5 mg/dL.

Let *r* be the component of interest, with component function *f*_*r*_ estimated by the tree-based GAM defining a set of thresholds *t*_*r*_ and associated outcome odds *y*_*r*_. We calculate slopes:

5 $${s}_{r,j}=\frac{{y}_{r,j+1}-{y}_{r,j}-1}{{t}_{r,j+1}-{t}_{r,j-1}}$$

and perform changepoint detection^6^ on the signs of *s*_*r*_. This statistic estimates the likelihood of a change in the direction of the component function at each threshold; the bootstrap provides confidence intervals. Finally, in our experiments, we restrict the search to only concave non-monotonicities because convex risk curves are usually explainable as healthy states (Fig. 7).

## Supplementary information

Supplementary Information

## Data Availability

All MIMIC datasets analyzed in this study are distributed freely under the PhysioNet platform^23^. The Pneumonia dataset analyzed in this study, as a motivating example, is available from the corresponding author on reasonable request. Intermediate data generated for analyses are available in the Python notebooks linked below.

## Code availability

Python notebooks reproducing these analyses, and utilities for automated search and plotting of discontinuities and non-monotonicities are available at: github.com/AdaptInfer/DeathByRoundNumbers.
