# Supplementary material for: The hidden risk of round numbers and sharp thresholds in clinical practice
Source: NPJ Digit Med. 2025 Nov 21;8:711. doi: 10.1038/s41746-025-02079-y (PMC12638946; doi:10.1038/s41746-025-02079-y)
Supplement: Supplementary file 1 — Supplementary Information [file 41746_2025_2079_MOESM1_ESM.pdf]

Supplementary Note 1 Supplementary Information

Supplementary Note 1.1 Simulation Experiments

To simulate the effects of confounding by treatment effects, we modeled intrinsic risk of a risk factor as $P(Y|X) = \sigma(f(x))$, where $f(x) = 2*(x-0.5)^2$, where σ is the logistic function. This simulates a healthy region surrounded by two unhealthy regions, with risk continuously increasing as the biomarker value moves away from the healthy range. We sample $x \in [0.35, 1.0]$, and apply $f(x)$ to generate an empirical set of risks R. We model four different types of treatments to generate treated risks $g(x)$:

- **Treatment Flattens Risk**: $g(x) = P_{50}(R)$
- **Treatment Caps Risk**: $g(x) = \min(f(x), P_{50}(R))$
- **Treatment Reduces Biomarker**: $g(x) = f(x - 0.2)$
- **Treatment Has Constant Benefit**: $g(x) = f(x) - P_{75}(R)$

We simulate two versions of treatment probabilities:

- **Strict Adherence**: $h(x) = \frac{1}{1+\exp(-500(x-\mu))}$
- **Loose Guidance**: $h(x) = \frac{1}{1+\exp(-20(x-\mu))}$

The threshold μ of the treatment protocol is determined by whether the threshold should be "optimal" ($\mu = \mu_0 = \text{argmin}_x\, f(x) < g(x)$), "too low" ($\mu = \mu_0 - 0.2$), or "too high" ($\mu = \mu_0 + 0.2$). The parameter values (-500 and -20) were chosen heuristically to illustrate extremes of adherence; alternate values yield qualitatively similar results. These treatment probabilities are visualized in Supplementary Figure 1.

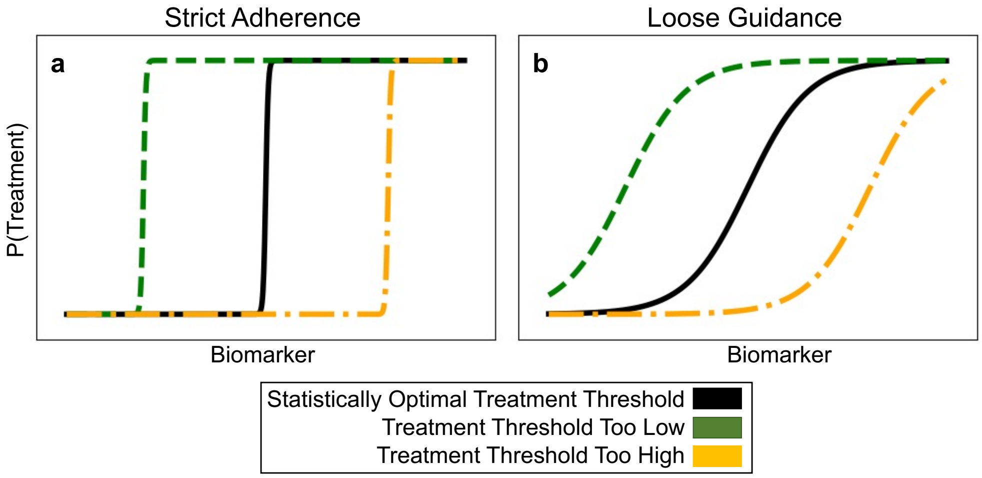

Supplementary Figure 1: Simulated treatment probabilities. **(a)** Treatment probabilities which follow strict adherence to a threshold-based protocol. **(b)** Treatment probabilities which treat the threshold-based protocol as loose guidance.

Given a treatment risk function and a treatment protocol function, we simulate observed population risk by $q(x) = f(x)(1 - h(x)) + g(x)h(x)$. Effects of hyperparameters do not change the fundamental characteristic shapes; code to reproduce these results are available at `github.com/AdaptInfer/DeathByRoundNumbers`.

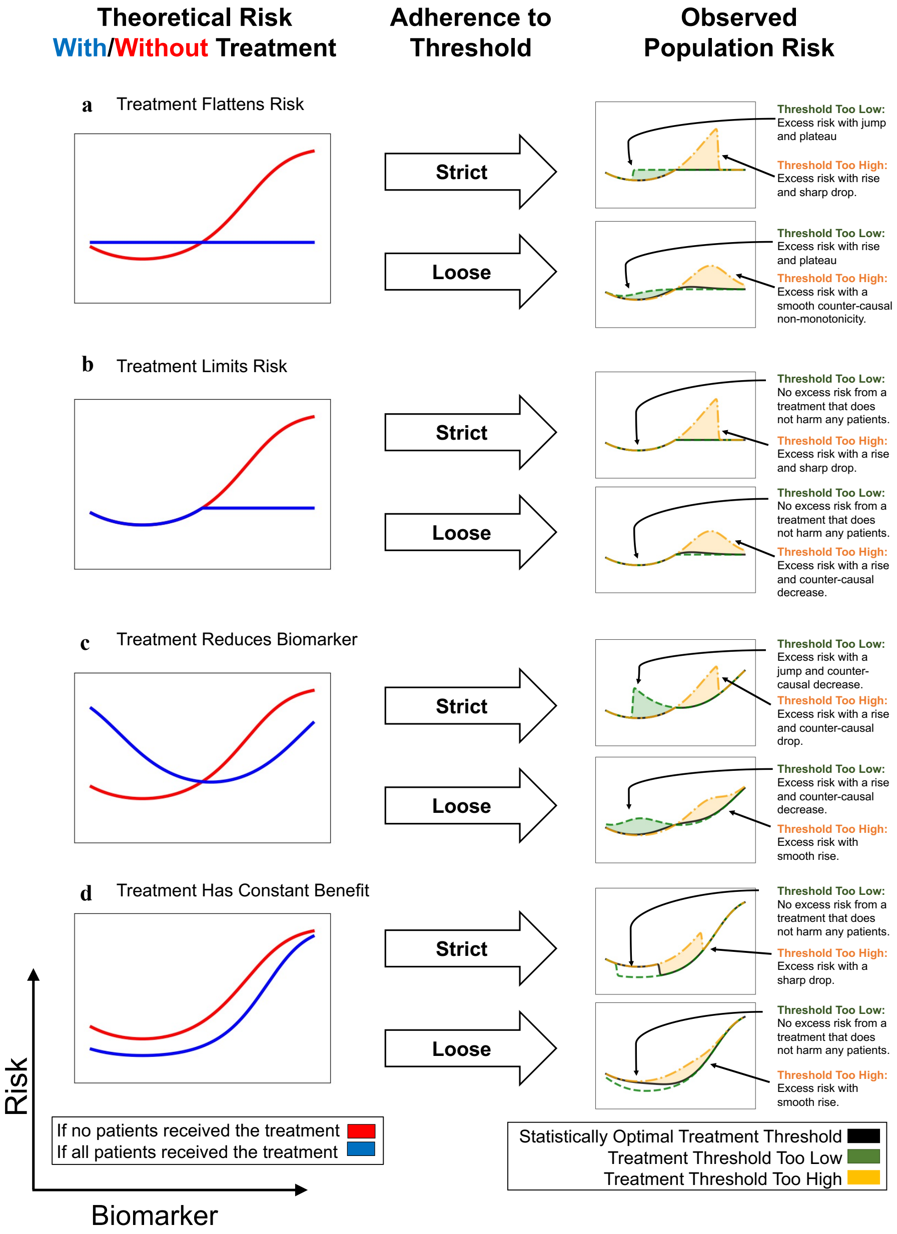

Supplementary Figure 2: Confounding from treatment effects produces characteristic shapes in population risk. In each row, we depict the underlying intrinsic risk for treated and untreated patients (left), adherence of treatment decisions to a threshold (middle), and observed risk (right). The optimal treatment threshold is the point where untreated risk crosses above treated risk; when treatment protocols are misaligned from optimality, observed risk includes excess risk. **(a)** When treatment induces a constant risk (e.g. surgery), and the treatment threshold is lower than optimal (green), there is a rapid rise in risk followed by a plateau. Conversely, if the treatment threshold is higher than optimal (yellow), there is an overshoot and drop. **(b)** When treatment caps risk (e.g bronchodilators), then no threshold is too low, while a high threshold produces a smooth increase in risk followed by a drop. **(c)** When treatment reduces the biomarker value (e.g. vasodilators reduce blood pressure), a low threshold produces a rapid and/or discontinuous rise in risk followed by a counter-causal non-monotonicity, while a high threshold produces a smooth increase in risk followed by a rapid and/or discontinuous decrease in risk. **(d)** If the treatment induced a constant benefit for all patients (e.g. an idealized treatment), then there is no threshold that is too low, and any threshold produces a counter-causal drop.

Supplementary Note 1.2 MIMIC

Preprocessing To ensure consistency across the MIMIC-II, MIMIC-III, and MIMIC-IV datasets, we implemented a standardized preprocessing pipeline. We restricted the analysis to ICU admissions and selected only risk factors present in at least two of the three datasets. Irrelevant columns, such as patient identifiers and administrative data, were excluded. Outliers in continuous variables, particularly lab values, were clipped to the 1st and 99th percentiles. Missing data were imputed with -1 for numeric variables and "Missing" for categorical features, ensuring consistency across datasets. In MIMIC-III and MIMIC-IV, we extract the worst value from the 1st 24 hour of admission for any multipy-recorded values. Additionally, MIMIC-IV temperature data, recorded in Fahrenheit, were converted to Celsius for standardization. These preprocessing steps harmonize the datasets, enabling direct comparisons. A detailed summary of patient characteristics after preprocessing is provided in Supplementary Table 1 and show similar patient characteristics across datasets.

Measurement	MIMIC-II	MIMIC-III	MIMIC-IV
Platelet	nan ± nan	237.1 ± 131.2	210.4 ± 109.0
SBP	119.6 ± 30.6	157.4 ± 25.0	121.8 ± 26.5
BUN	26.8 ± 21.9	28.6 ± 23.4	26.2 ± 21.9
Chloride	nan ± nan	103.3 ± 6.7	103.8 ± 6.6
Temp	36.9 ± 0.9	37.8 ± 0.8	36.7 ± 1.8
Magnesium	nan ± nan	2.0 ± 0.5	2.0 ± 0.4
HeartRate	88.5 ± 19.6	112.1 ± 21.9	nan ± nan
Creatinine	nan ± nan	1.5 ± 1.7	1.4 ± 1.5
Bilirubin	1.2 ± 2.7	nan ± nan	1.7 ± 3.8
WBC	12.0 ± 11.8	12.8 ± 10.0	11.8 ± 7.2
Age	63.2 ± 17.5	63.2 ± 16.1	64.7 ± 16.9
Sodium	138.3 ± 4.9	138.0 ± 5.3	138.3 ± 5.2
Potassium	4.3 ± 0.9	4.2 ± 0.8	4.2 ± 0.7
Glucose	nan ± nan	152.6 ± 94.9	142.5 ± 72.7
Albumin	nan ± nan	3.1 ± 0.5	3.1 ± 0.7

Supplementary Table 1: Patient characteristics using features shared across all three MIMIC versions, including the mean ± standard deviation of each feature. NaN indicates that the feature was not recorded in that dataset. Patient characteristics are relatively consistent across datasets.

Accuracy of GAM In general we find that GAMs trained with EBMs provide a sweet spot for modeling healthcare data because the models are complex enough to be very accurate and represent high-resolution details such as discontinuities while still remaining fully interpretable. Moreover, because the models are restricted in complexity, they are much less likely to learn spurious effects that do not generalize to held-out examples. Finally, `InterpretML` uses bootstrap resampling [21] to reduce variance and make the learned models easier to interpret, and also to estimate confidence intervals that help distinguish true from spurious detail in the learned response curves. We benchmark the model against several baselines (Supplementary Table 2) to examine the performance of GAMs with EBMs. As baselines, we include 3 versions of XGBoost [24] which differ in the max depth (XGB-1 creates trees of depth 1, XGB-2 creates trees of depth 2, and XGB-3 creates trees of depth 3). As a result, XGB-1 is a GAM which can be directly compared against the EBM GAM, and the performance of the deeper XGBoost versions can be compared to evaluate the utility of permitting interaction effects in the model. Finally, we also benchmark against fully-connected neural networks (MLP). In all four of these datasets, the EBM performs as well or better than the baseline methods.

We note that while XGBoost is often optimized over a wide hyperparameter grid, performance across these datasets was already maximized at shallow depths (1–2), with deeper models (depth=3) performing worse. Thus, a fully optimized XGBoost model would converge to the results reported here, and no additional benefit beyond pairwise interactions was observed.

Dataset	Model	AUROC	AUPRC	F1-Score
Pneumonia	XGB-1	0.86 ± 0.01	0.47 ± 0.01	0.34 ± 0.01
	XGB-2	0.84 ± 0.01	0.43 ± 0.02	0.34 ± 0.01
	XGB-3	0.80 ± 0.04	0.39 ± 0.02	0.35 ± 0.03
	MLP	0.75 ± 0.05	0.29 ± 0.02	0.22 ± 0.03
	EBM	$\mathbf{0.88 \pm 0.03}$	$\mathbf{0.53 \pm 0.02}$	$\mathbf{0.37 \pm 0.02}$
MIMIC-II	XGB-1	0.78 ± 0.02	0.35 ± 0.02	0.18 ± 0.01
	XGB-2	0.78 ± 0.02	0.35 ± 0.02	0.18 ± 0.02
	XGB-3	0.77 ± 0.05	0.35 ± 0.02	$\mathbf{0.28 \pm 0.02}$
	MLP	0.74 ± 0.04	0.30 ± 0.03	0.07 ± 0.02
	EBM	$\mathbf{0.79 \pm 0.02}$	$\mathbf{0.38 \pm 0.03}$	0.17 ± 0.02
MIMIC-III	XGB-1	0.73 ± 0.02	0.26 ± 0.01	0.07 ± 0.00
	XGB-2	0.72 ± 0.04	0.23 ± 0.02	0.11 ± 0.00
	XGB-3	0.69 ± 0.05	0.22 ± 0.05	$\mathbf{0.14 \pm 0.01}$
	MLP	0.68 ± 0.08	0.24 ± 0.03	0.03 ± 0.01
	EBM	$\mathbf{0.76 \pm 0.01}$	$\mathbf{0.31 \pm 0.01}$	0.06 ± 0.01
MIMIC-IV	XGB-1	0.74 ± 0.01	0.24 ± 0.01	0.10 ± 0.03
	XGB-2	$\mathbf{0.75 \pm 0.03}$	$\mathbf{0.27 \pm 0.03}$	0.17 ± 0.03
	XGB-3	0.73 ± 0.05	0.25 ± 0.03	$\mathbf{0.20 \pm 0.02}$
	MLP	0.73 ± 0.02	0.25 ± 0.02	0.13 ± 0.01
	EBM	$\mathbf{0.75 \pm 0.03}$	0.26 ± 0.01	0.08 ± 0.01

Supplementary Table 2: Accuracy metrics of the predictions made by EBM and other models on the held-out test set of patients demonstrate that EBM is near or better-than comparable general-purpose models on these datasets. Values are the (mean± std) over 5 experimental runs. XGBoost performance is shown for varying max-depths to assess the benefit of modeling interactions explicitly. Performance did not improve with higher complexity (depth=3), suggesting minimal benefit beyond pairwise interactions.

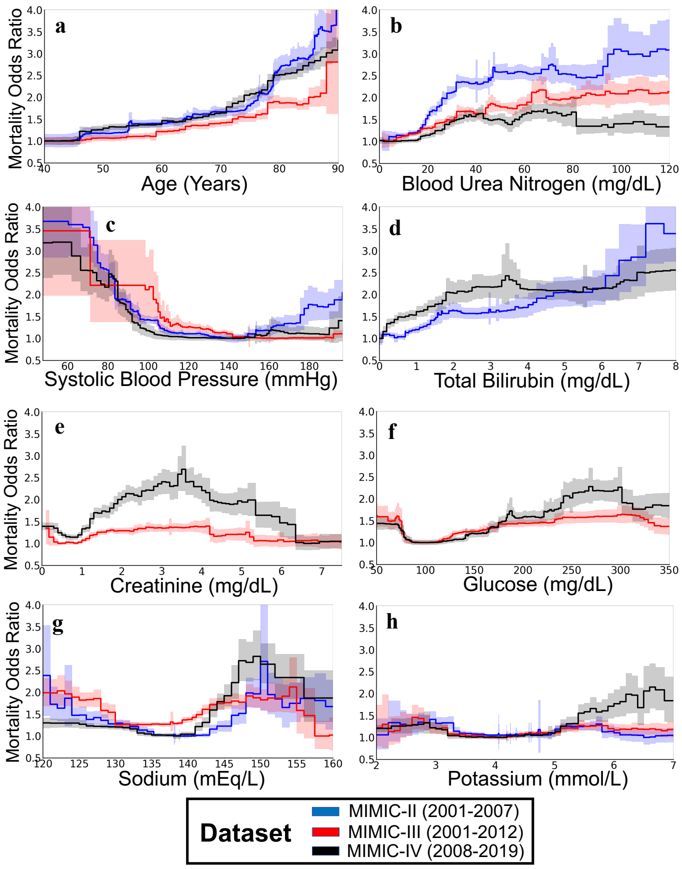

Supplementary Figure 3: The effects of thresholds are visible in mortality risk curves for patients in ICUs. In all plots, we show the risk associated with each risk factor after correcting for all other observable factors of patient risk using a generalized additive model (with 95% confidence intervals shaded). The three datasets span three decades of intensive care at a single hospital system. The impacts of creatinine, sodium, age, and blood urea nitrogen are studied in detail in the main text. **(a-d)** Biomarkers that display discontinuous impacts of treatment effects, including round-number ages, blood urea nitrogen at 45mg/dL, systolic blood pressure below 100 mmHg, and Bilirubin above 2mg/dL. **(e-h)** Biomarkers which display counter-causal impacts of treatment effects, including elevated creatinine above 6mg/dL corresponding to reduced mortality risk, elevated glucose above 250 or 300 mg/dL corresponding to reduced mortality risk, hypernatremia above 150 mEq/L corresponding to reduced mortality risk (and in the oldest dataset, severe hyponatremia below 120 mEq/L also corresponded to reduced mortality risk), and elevated potassium (above 6 mmol/L) corresponding to increased mortality risk only in the newest dataset.

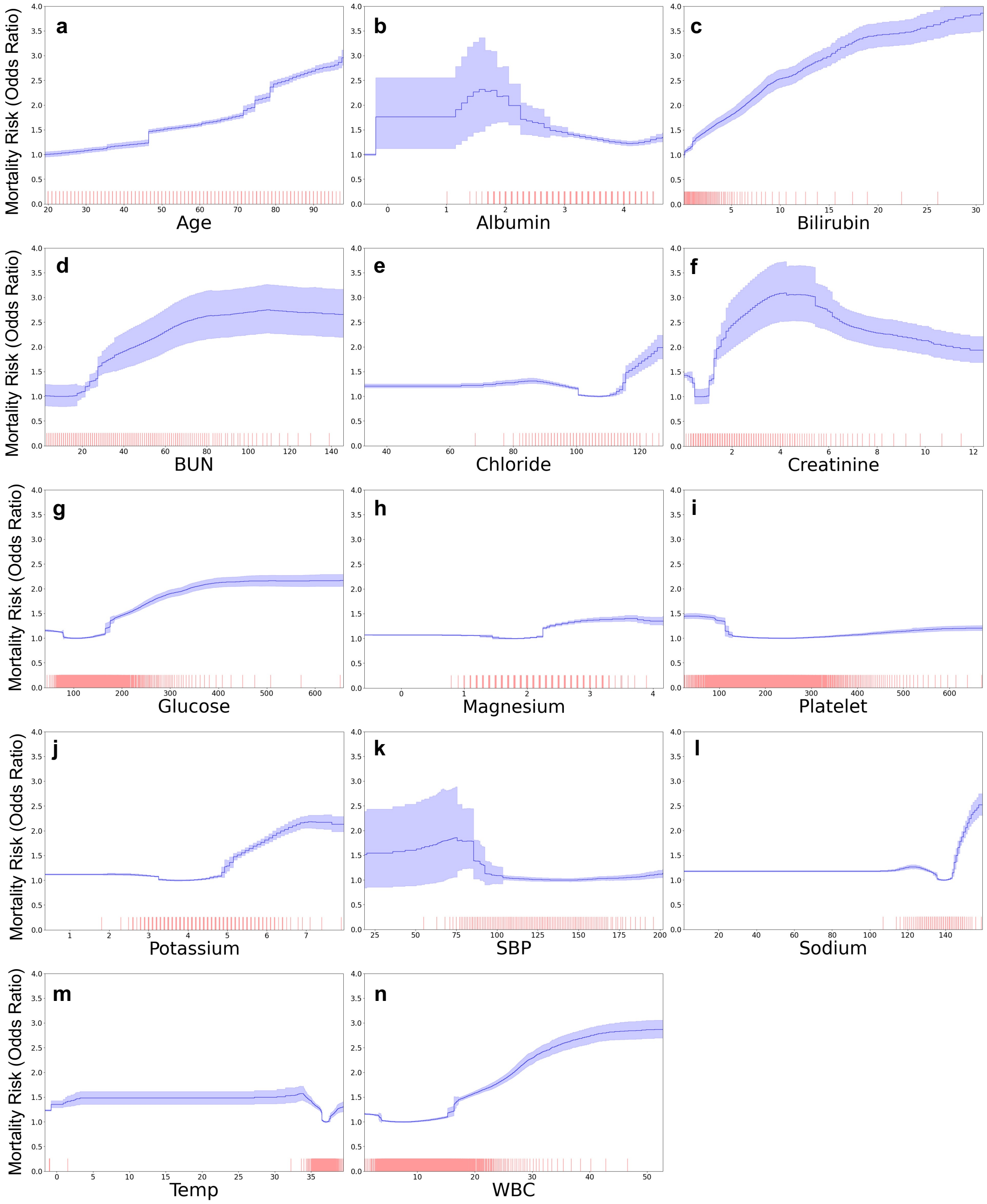

Supplementary Figure 4: Uncorrected (marginal) risks of mortality in MIMIC-IV. In each plot, the blue curve shows the risk associated with each factor without correcting for other observable patient variables, and red ticks along the horizontal axis denote 10 patients. Features shown are: **(a)** age, **(b)** albumin, **(c)** bilirubin, **(d)** blood urea nitrogen, **(e)** chloride, **(f)** serum creatinine, **(g)** glucose, **(h)** magnesium, **(i)** platelet count, **(j)** potassium, **(k)** systolic blood pressure, **(l)** sodium, **(m)** temperature, **(n)** white blood cell count. These curves show uncorrected risks for illustrative comparison; corrected risks that account for other variables are estimated directly by the generalized additive model presented in the main text.

Anomaly Type	Feature	Value
Non-Monotonicity	SBP	81.0
	Chloride	115.0
	Creatinine	3.5
	Magnesium	2.6
	Platelet	16.0
	Potassium	6.7
	Temp	35.0
Discontinuity	Glucose	106
	Glucose	99
	Age	50
	Age	78
	SBP	110
	Glucose	122
	Age	90
	SBP	119
	Platelet	247
	Glucose	174
	Glucose	117
	Platelet	204
	Glucose	85
	Age	45
	Glucose	144
	Chloride	74
	Glucose	128
	Platelet	127
	Sodium	134
	Age	35
	Platelet	90
	Age	55
	Platelet	230
	WBC	4
	Platelet	179
	Platelet	144
	Age	27
	Sodium	138

Supplementary Table 3: All anomalies found in MIMIC-IV by automated tests. Hyperparameters used were: for non-monotonicities, probability threshold=0.1; for discontinuities, minimum samples=2000 and minimum effect size=1.1.
